# The influence of internal models on feedback-related brain activity

**DOI:** 10.3758/s13415-020-00820-6

**Published:** 2020-08-18

**Authors:** Franz Wurm, Benjamin Ernst, Marco Steinhauser

**Affiliations:** Catholic University of Eichstätt-Ingolstadt, Ostenstraße 27, 85072 Eichstätt, Germany

**Keywords:** Event-related potentials, Feedback processing, Model-free learning, Model-based learning, Reinforcement learning, Time-frequency analysis

## Abstract

Decision making relies on the interplay between two distinct learning mechanisms, namely habitual model-free learning and goal-directed model-based learning. Recent literature suggests that this interplay is significantly shaped by the environmental structure as represented by an internal model. We employed a modified two-stage but one-decision Markov decision task to investigate how two internal models differing in the predictability of stage transitions influence the neural correlates of feedback processing. Our results demonstrate that fronto-central theta and the feedback-related negativity (FRN), two correlates of reward prediction errors in the medial frontal cortex, are independent of the internal representations of the environmental structure. In contrast, centro-parietal delta and the P3, two correlates possibly reflecting feedback evaluation in working memory, were highly susceptible to the underlying internal model. Model-based analyses of single-trial activity showed a comparable pattern, indicating that while the computation of unsigned reward prediction errors is represented by theta and the FRN irrespective of the internal models, the P3 adapts to the internal representation of an environment. Our findings further substantiate the assumption that the feedback-locked components under investigation reflect distinct mechanisms of feedback processing and that different internal models selectively influence these mechanisms.

## Introduction

In our everyday life, decision making is usually accompanied by uncertainties. To resolve these uncertainties, a variety of informational cues can guide behavior. Past experience with decision outcomes can act as a valuable and straightforward criterion that indicates whether the decision maker should repeat or switch actions. For instance, if the last meal at a specific restaurant was of poor quality, maybe one should consider changing the restaurant next time. However, the history of past decision outcomes is not the only cue that can guide decision making. For example, internal models based on explicit knowledge, such as reviews on a restaurant`s quality, may serve as a good proxy for costly experience and thus can facilitate the optimization of decision making. It is assumed that these two sources of information—past experience and internal models—improve decision making via two different learning mechanisms called model-free learning and model-based learning (Daw & O’Doherty, 2014; Dayan & Berridge, 2014; O’Doherty, Cockburn, & Pauli, 2017). Despite the long held assumption of computationally dissociable learning mechanisms, recent literature suggests an integration of model-free and model-based information at the level of feedback processing (Daw, Gershman, Seymour, Dayan, & Dolan, 2011; Sambrook, Hardwick, Wills, & Goslin, 2018), and this integration might be sensitive to the structure of the environment as represented by an internal model (Eppinger, Walter, & Li, 2017; Lee, Shimojo, & O’Doherty, 2014). We ask how different environmental structures and thus internal models exert influence on both behavioral and neural aspects of feedback evaluation. We contrasted environments with predictable and random stage transitions in a multistage task and investigated which neural correlates of feedback processing in event-related potentials (ERPs) and oscillatory activity are sensitive to differences between the involved internal models.

Learning from feedback has typically been formalized within the reinforcement learning framework, which assumes that stimulus-response reward associations are acquired and updated on a trial-by-trial basis to flexibly guide behavior (Sutton & Barto, 1998). Current psychological theories propose that not only a single but two (or more) qualitatively distinct families of reinforcement learning mechanisms are at hand to guide choice behavior (Dayan & Niv, 2008). *Habitual* or *model-free reinforcement learning mechanisms* learn to choose between actions by, first, assigning values to actions based on their past history of reward and punishment, and then, deciding for the action with the highest value. To validly estimate the value of an action, a so-called reward prediction error (RPE) is calculated. RPEs reflect the difference between an action`s value estimate from past experience and the actual outcome of the action on the given trial and therefore provide the decision maker with an instrumental teaching signal to optimize behavior. By incrementally integrating RPEs in the estimated value of a chosen action, outcome expectations are formed in a retrospective manner, thus allowing for an experience-driven behavioral adaptation. However, because expectations derived from RPEs fail to establish an explicit representation of environmental contingencies, the decision maker often behaves inaccurately in complex environments (Dayan & Niv, 2008; Doll, Simon, & Daw, 2012). This disadvantage of model-free mechanisms is overcome by *goal-directed* or *model-based reinforcement learning mechanisms*, which are able to prospectively form expectations based on explicit knowledge about environmental contingencies. Numerous sources of explicit knowledge can be identified including learning from instructions to mere observation without feedback, or deliberate reasoning. Crucially, these model-based mechanisms generate an internal (world) model that allows for deriving predictions about future states of the external world (Daw, Niv, & Dayan, 2005; Dickinson & Balleine, 2002; Doya, 1999; Gläscher, Daw, Dayan, & O’Doherty, 2010; Tolman, 1948), further improving adaptivity beyond model-free learning.

On a behavioral level, evidence for model-based learning primarily comes from studies using multistage decision problems, such as the Markov decision task (Daw, Gershman, Seymour, Dayan, & Dolan, 2011). In a classic version of the task, participants are faced with a decision between two stimuli at a first decision stage. With a given set of transition probabilities (the *transition structure*), each decision at the first stage leads to two distinct stimulus pairs presented at a second decision stage. More specifically, each first-stage decision is linked with one of the stimulus pairs with a high probability (common transition) and with the alternative stimulus pair with a low probability (rare transition). Depending on the participants’ second-stage decision, feedback about a monetary reward or loss is delivered. Each of the stimuli at the second stage is associated with a distinct reward probability that changes over time (the *reward structure*). Crucially, model-based mechanisms and model-free mechanisms differ with respect to how information about the probability of a transition between first-stage decisions and second-stage stimuli is incorporated for optimizing first-stage decisions. On the one hand, the model-free mechanisms adapt their behavior only with respect to past outcomes but regardless of whether that past outcome followed a common or rare transition between first-stage decision and second-stage stimuli. Irrespective of the probability of the experienced transition, a reward after the second stage would reinforce the first-stage decision and thus increase the probability that the same first-stage decision is made again, thus leading to stay behavior. On the other hand, the model-based mechanisms show the emergence of an interactive pattern between past outcome and transition. After a common transition, reward would reinforce the first-stage decision in the same way as in model-free learning and thus would lead to stay behavior. In contrast, a reward after a rare transition would reinforce the first-stage decision that would lead to the same outcome via the common transition, thus increasing the probability of the alternative first-stage decision leading to switch behavior. The predicted behavioral patterns of these two families of learning mechanisms, which culminate in a main effect of outcome (model-free) and an interaction between outcome and transition (model-based) have been repeatedly confirmed in humans (Daw et al., 2011; Doll, Bath, Daw, & Frank, 2016; Doll, Duncan, Simon, Shohamy, & Daw, 2015; Gläscher et al., 2010; Lee et al., 2014; Wunderlich, Smittenaar, & Dolan, 2012).

To investigate the neural correlates of model-free and model-based reinforcement learning, EEG can be used to precisely track processes at the different stages of the Markov decision task. When it comes to feedback processing, separable time-domain and frequency-domain components have already been identified to play a major role in reinforcement learning and the formation of expectations: In the time-domain, the feedback-related negativity (FRN) which is a fronto-central component that occurs between 200 and 350 ms after feedback presentation is hypothesized to reflect an RPE (Chase, Swainson, Durham, Benham, & Cools, 2011; Holroyd & Coles, 2002; Sambrook & Goslin, 2015). It is typically measured as a negative deflection following negative outcomes relative to positive outcomes: the so-called FRN effect. Although still a matter of debate (Sambrook & Goslin, 2016; Sambrook et al., 2018), the FRN is suggested to be modulated by model-free expectations (see San Martín, 2012; Walsh & Anderson, 2012). A further component related to expectancy is the P3, which is hypothesized to be involved in the updating of working-memory representations (Polich, 2007). The P3 is a parietal and positive going component that occurs around 300-600 ms after stimulus presentation. When elicited by feedback, the P3 has been shown to be associated with expectancy modulations, i.e., larger amplitudes following unexpected outcomes (Bellebaum & Daum, 2008; Hajcak, Holroyd, Moser, & Simons, 2005; Hajcak, Moser, Holroyd, & Simons, 2007; but also see Wu & Zhou, 2009). Because it has been proposed to be associated with the updating of an internal world model in working memory (Donchin & Coles, 1988; Nieuwenhuis, Aston-Jones, & Cohen, 2005; but see Rac-Lubashevsky & Kessler, 2019), the P3 could be viewed as being closely linked to model-based reinforcement learning.

In addition to these ERPs, neural oscillations have been shown to reflect specific aspects of feedback processing. Theta band activity (4-8 Hz) at frontocentral electrodes has been suggested to communicate the need for cognitive control and behavioral adaptation (Cavanagh & Frank, 2014; Cavanagh & Shackman, 2015). With its spatial distribution similar to the FRN (e.g., Cavanagh, Figueroa, Cohen, & Frank, 2012), fronto-central theta has also been found to reflect the evaluation of primary stimulus features such as outcome valence and salience (Bernat, Nelson, & Baskin-Sommers, 2015) as well as RPEs (Cavanagh, Frank, Klein, & Allen, 2010). A further frequency band that has been found to reflect RPEs (Cavanagh, 2015) is the delta band (1-4 Hz). In contrast to fronto-central theta, centro-parietal delta activity might reflect the assessment of higher-order secondary stimulus features, such as relative outcome (Bernat et al., 2015). Taken together, while theta and delta activity appear to reflect separable cognitive processes and contribute differentially to the ERP waveforms (Bernat et al., 2015; Cavanagh, 2015), the relationship between frequency components, ERP components, and the underlying mechanisms is still unclear and a matter of ongoing research.

**Fig. 1:**
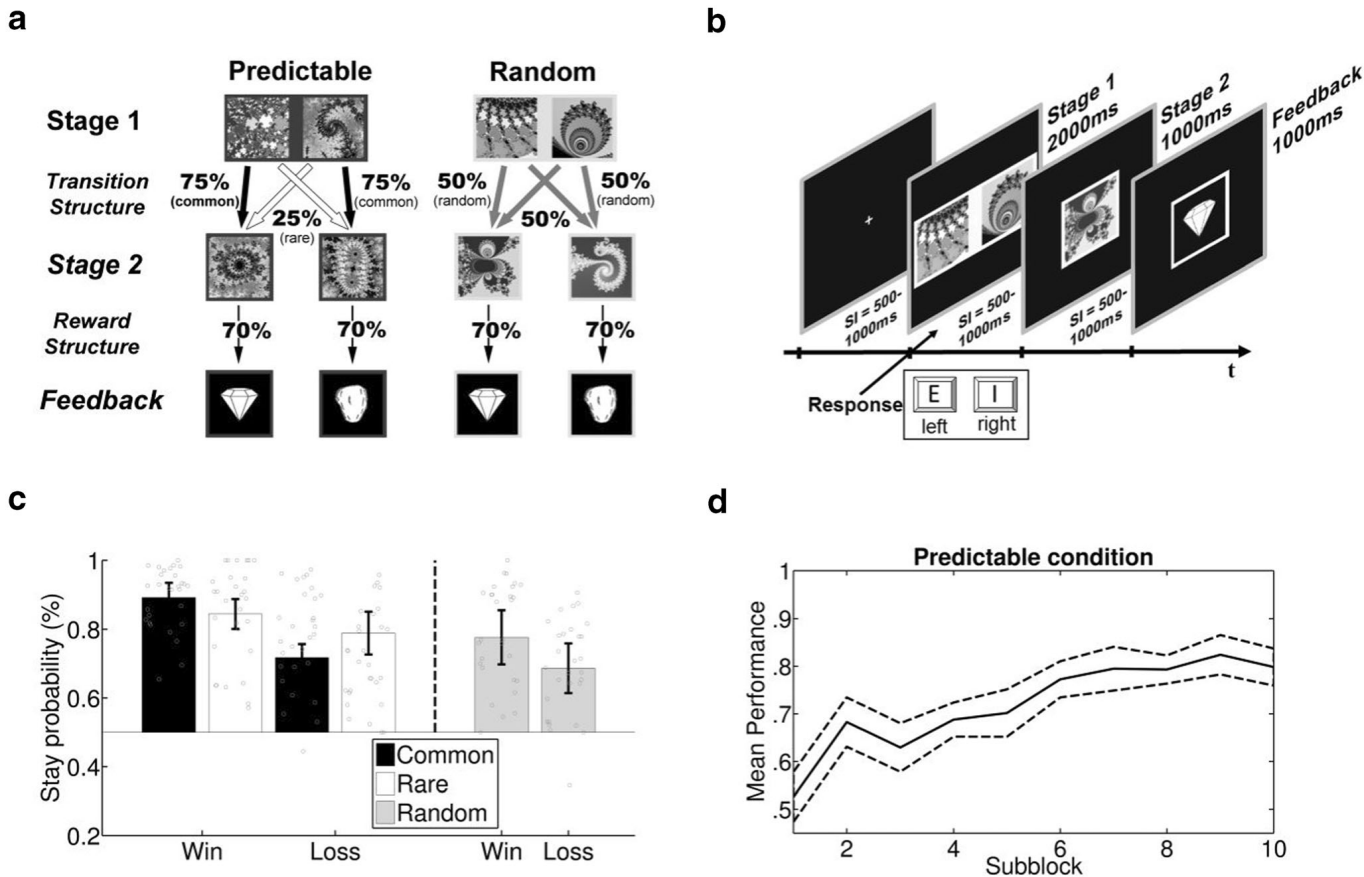
**a. **Schematic representation of the environmental contingencies for the predictable and random conditions**.** The conditions differed regarding their transition structure but had an identical reward structure. **b.** Graphical illustration of a trial: After fixation cross presentation, participants had to decide between two pictures at Stage 1 and were subsequently forwarded to Stage 2. Depending on the second-stage stimulus, feedback was presented. **c.** Stay probabilities, averaged across subjects. Error bars depict ±SEM. Gray circles indicate stay probabilities for the individual subjects. **d.** Subjects’ performance in predictable conditions, plotted as the mean proportion of correct decisions across subblocks. Correct decisions are defined as first-stage choices for the stimulus which commonly led to the high reward second-stage picture. Subblocks were assigned post-hoc by separating the 50 trials of the predictable condition in each block into ten equal parts, consisting of 5 trials. Dashed lines depict ±SEM

The goal of the present study was to investigate the influence of distinguishable internal models of the environmental structure on the neural correlates of feedback processing. To allow for the emergence of two distinguishable internal models, we contrasted two different transition structures in a two-stage Markov decision task (Eppinger et al., 2017; Lee et al., 2014). In the predictable condition, transition probabilities were highly differentiated between common and rare transitions (75% vs. 25%; see Fig. 1A, black and white arrows), leading to an easily predictable transition structure. We hypothesized that such a predictable transition structure should favor the formation of an equally predictable internal world model, which can be used to adapt first-stage decisions on a trial-to-trial basis. In the random condition, transition probabilities were fixed at chance level (50%; see Fig. 1A, gray arrows), resulting in a random (i.e., unpredictable) transition structure. We hypothesized that this should favor the formation of an internal world model that promotes stochastic (i.e., random) behavior, thus counteracting trial-to-trial adaptation of first-stage decisions.^1^ Crucially, despite their differing transition structure, both task conditions had an identical reward structure. That is, the conditional reward probability given a specific second-stage stimulus was the same across both conditions. This allowed us to investigate effects of reward expectancy on feedback processing in both conditions without confounding differences in these objective conditional reward probabilities.

Our central question was which neural correlates of feedback processing are sensitive to the type of internal model. We first investigated stay/switch behavior in the two conditions. While the predictable condition should reveal the usually obtained signatures of model-based and model-free control, an important question was whether we find evidence for behavioral adaptation in the random condition. Although the random condition does not allow for separating the contribution of model-free and model-based control, demonstrating an effect of reward on subsequent stay probabilities in this condition would show that feedback is utilized for behavioral adaptation even when this feedback cannot be used to improve first-stage decisions. We then analyzed feedback-related activity in the time and frequency domain to investigate which components are sensitive to the type of internal model. In addition to valence effects, we focused on expectancy effects (based on conditional reward probabilities) as these indicate whether activity is related to an RPE. We expected to find reduced valence and expectancy effects in the random compared with the predictable condition for components sensitive to a model-based feedback evaluation. Because the random internal model promotes stochastic behavior rather than a feedback-based adaptation of first-stage decisions, components involved in model-based feedback evaluation should be attenuated in the random condition. In a second part, we fit computational models to the data, which explicitly implemented the ideas of a random and predictable internal model. Crucially, these models allowed for deriving trial-wise RPE estimates, which could then be used to inform EEG data analysis. By correlating these RPEs with single-trial measure of feedback-related activity separately for the random and predictable conditions, we obtained a complementary measure of the strength by which each component is involved in processing feedback in these conditions.

## Material and methods

### Participants

Thirty-seven participants (30 females) between 19 and 33 years of age (*M* = 23.00, *SD* = 3.49) with normal or corrected-to-normal vision participated in the study. Participants were recruited at the Catholic University of Eichstätt-Ingolstadt and received course credit for participation and a performance-dependent bonus (*M* = 0.55 €). For the analyses, seven participants were excluded due to excessive EEG artifacts, and one participant was excluded due to low performance in set identification (for further details, see below). Taken together, 29 participants (24 females) between 19 and 33 years of age (*M* = 22.69, *SD* = 3.70) entered the analyses. All participants provided informed consent and the study protocol was approved by the ethical committee of the Catholic University of Eichstätt-Ingolstadt.

### Stimuli

The stimuli consisted of 32 greyscale-normalized quadratic fractals derived from a Mandelbrot set (for examples, see Fig. 1A). Pictures were converted into 200 x 200 pixel images with a side length of 4.45° visual angle at a viewing distance of 70 cm. Before the experiment, all pictures were grouped into eight sets with four stimulus pictures each. From each set, two randomly drawn pictures were assigned to be first-stage stimuli, and the remaining two were assigned to be second-stage stimuli. Half of the sets were assigned to the predictable condition and the other half to the random condition. Each picture was presented in a frame with the frame color (light gray or dark gray), indicating the condition (predictable or random). Frame colors and sets were assigned randomly for each block. The resulting stimuli had a side length of 5.16° visual angle. All stimuli were presented on a black background.

### Task and procedure

A crucial goal of this study was to investigate the effects of feedback expectedness on feedback-related brain activity in two conditions (random vs. predictable) that a) differ in the involved internal model, but b) are associated with comparable feedback probabilities. To achieve this, we modified the classical two-stage Markov decision task in the following way (Figure 1A). Participants first chose between two first-stage stimuli. The *transition structure* determined the probabilities by which a specific second-stage stimulus could occur. As in a previous study (Gillan, Otto, Phelps, & Daw, 2015), this second-stage stimulus consisted of a single picture and no further decision was required at the second stage. Feedback was delivered only on the basis of this second-stage stimulus. The probability of positive and negative feedback was determined by a *reward structure* that was identical between random and predictable conditions and remained constant over time. Trials from the random and predictable conditions were randomly intermixed, and participants had to find out themselves which stimuli were associated with each condition. This was done to prevent systematic strategic differences (e.g., ignoring feedback in the random condition) and thus to make the two conditions as similar. The analogous reward structure in the two conditions ensured that differential expectancy effects on feedback-related brain activity are not related to changing objective reward probabilities (as in the classical Markov decision task) but can be traced back to the hypothesized differences in the involvement of internal models between conditions.

The random and predictable conditions differed only with respect to the transition structure between the first and the second stage (Figure 1A). Choosing a stimulus from the two first-stage stimuli implied that each of the two possible second-stage stimuli appeared with a specific probability, and this probability differed between predictable and random conditions. In the predictable condition, one of the two first-stage stimuli was linked to one second-stage stimulus with a probability of 75% (common transition; black arrow in Fig. 1A) and to the other second-stage stimulus with a probability of 25% (rare transition; white arrow in Fig. 1A), and these probabilities were reversed for the other first-stage stimulus. In the random condition, all transition probabilities between first-stage and second-stage stimuli were fixed at 50% (gray arrows in Fig. 1A). The reward structure between the second stage and the feedback was the same for predictable and random conditions. In each condition, one of the two second-stage stimuli led to a win with a probability of 70% (high reward stimulus) whereas the other second-stage stimulus led to a loss with a probability of 70% (low reward stimulus). Crucially, this implies that although predictable and random conditions differed with respect to the transition structure (probability of second-stage stimulus given first-stage decision), the reward structure (probability of feedback given second-stage stimulus), and thus the expectedness of feedback was the same across conditions.

The procedure of a trial is illustrated in Fig. 1B. At the beginning of each trial, one of the two conditions (predictable vs. random) was randomly chosen. First, a fixation cross was shown for a random and exponentially distributed interval ranging between 500 and 1,000 ms. Then, the first-stage stimuli of the chosen condition were presented for 2,000 ms in counterbalanced order (left, right). During this time, participants had to make a choice by either pressing the “E” (left) or “I” (right) key of a standard keyboard using the index finger of the left or right hands. If no response had occurred before stimulus offset, the trial was aborted and a miss feedback was presented until participants continued by pressing the space key. If a response had occurred, another fixation cross was presented for a random interval between 500 and 1,000 ms. Then, the second-stage stimulus was presented for 1,000 ms. The second-stage stimulus was chosen based on the participants’ first-stage decision and the condition-specific transition structure. After the presentation of the second-stage stimulus, again a fixation cross was displayed for 500 to 1,000 ms, followed by one of two feedback stimuli for 1,000 ms. Feedback was chosen based on the reward structure explained above. Diamonds indicated wins (+3 ct), whereas stones indicated losses (−3 ct). At the end of each trial a fixation cross, again displayed for 500 to 1,000 ms, led to a screen that informed participants whether a decision was made in the previous trial and participants were instructed to press the space key to continue to the next trial.

For each of the four blocks employed in the experiment, participants worked through 100 trials. Each of the sets associated with a condition (random vs. predictable) was presented equally often, resulting in 50 trials of the predictable condition and 50 trials of the random condition. The two conditions were randomly mixed with the constraint that one condition could only be presented by a maximum of three trials in a row. At the beginning of each block participants saw a screen providing a brief reminder of the most important task features (e.g., reward magnitudes, transition structure, and relevant keys). However, they were not instructed about which set was assigned to which condition (predictable vs. random). To familiarize participants with the abstract visual stimuli, all stimuli used in the upcoming block were presented randomly but arranged by set and stage. At the end of each block, participants were asked to judge (1) which set yielded more bonus and (2) which set had the predictable transition structure. The second question was used to exclude blocks from the EEG analysis in which participants did not correctly identify the predictable and random set.

Before starting the main experiment, participants were instructed and practiced the task by working through two practice blocks. In the first practice block, participants learned about the probabilistic reward structure via written instructions and by viewing 20 trials to demonstrate the probabilistic link between the second stage and the feedback stage. In the second practice block, participants learned about the probabilistic transition structure. Again, they received written instruction but completed 20 active practice trials, demonstrating the probabilistic link between the first and the second stage. Stimuli used in the practice blocks were not used in the main part of the experiment. In the instruction, the task was framed as a treasure hunting. Participants should imagine to be treasure hunters. Stimulus pairs at the first stage were instructed to be a treasure map with two possible routes that can be taken to search for treasures. On their way, natural disasters and monsters may lead the treasure hunter astray, which sometimes results in following the nonchosen route. Second stage stimuli were introduced as treasure chests which would contain or not contain treasure.

### Data acquisition and analysis

Behavioral data were analyzed using MatLab v8.6 (The Mathworks Inc., Natick, MA) and R (R Core Team, 2016). A RL model was implemented and fitted using R and STAN (Carpenter et al. 2017). ERP data were analyzed using custom-made routines in MatLab as well as EEGLAB 13.5.4b (Delorme & Makeig, 2004), an open source toolbox for EEG data analysis (EEGLAB toolbox for single-trial EEG data analysis, Swartz Center for Computational Neurosciences, La Jolla, CA; http://www.sccn.ucsd.edu/eeglab).

#### Behavioral data

In line with previous work (Daw et al., 2011; Gillan et al., 2015), behavioral data were analyzed using logistic regression with mixed-effect models. The predictable and random conditions were analyzed separately as transition type (common vs. rare) could be distinguished only for the predictable condition. For analyzing the predictable condition, we submitted reward type and transition type of the preceding trial *of the same condition* (here: predictable) as predictors to test for stay/switch behavior in the present trial. The reward type of the preceding trial could either be a *win* or a *loss* (coded as 1 and −1). The transition type could either be *common* or *rare* (coded as 1 and −1). The choice type could either be a *switch* or *stay* in behavior (coded as 0 and 1) depending on both the choice on the preceding trial of the same condition and the ongoing trial. For the random condition, the same analysis was used with the exception that transition type was omitted. Please note that, in both analyses, the preceding trial for the analysis was not necessarily the preceding trial in the experiment, because the random and predictable condition were presented in random sequence in a block. Finally, in both analyses, the within-subjects variables (intercept, main effects of reward and transition type, their interaction) were implemented as random effects and therefore were allowed to vary across participants (Daw et al., 2011; Gillan et al., 2015).

#### Computational modeling

Behavioral data were further analyzed using computational modeling. Whereas the regression analysis above takes only information from the last trial of the same condition into account, this model-based approach accounts for incremental effects of learning across several trials. Furthermore, the explicit implementation of the internal representations in our computational models allows for an in-depth assessment of how neural activity is related to specific aspects of learning such as RPEs. As described in previous studies (Daw et al., 2011; Eppinger et al., 2016; Gillan et al., 2015), we fitted participants’ choice behavior using a hybrid RL model, which can isolate the contributions of model-based and model-free mechanisms to individual behavior. Although we do not know whether model-based control is involved in the random condition, we also applied a hybrid architecture for this condition. Here, the “model-based” mechanism represents the chance level transition probabilities from the random condition. As a consequence, this mechanism introduces stochastic (i.e., random) choice behavior in the model which counteracts model-free control. Whether such a mechanism can be viewed as corresponding to model-based control is discussed in the discussion section. Because its computational implementation is equivalent to model-based control in the predictable condition, it is introduced as a model-based mechanism below which however should not imply any interpretation on the nature of this mechanism.

The *model-free mechanism* uses temporal difference learning to incrementally update stimulus values, *Q*_*MF* ∣ *S*2_ for observed picture *p*_*x*_ of trial *t* at the second stage according to the equation

$$ {Q}_{MF\mid S2}\left({p}_x,t+1\right)={Q}_{MF\mid S2}\left({p}_x,t\right)+\alpha \left[r(t)-{Q}_{MF\mid S2}\left({p}_x,t\right)\right], $$

where *α* is the learning rate and *r*(*t*) the reward received in that trial. The term in square brackets contains the RPE elicited by the feedback. Then, action values for the visited state-action pair *a* at the first stage, *Q*_*MF* ∣ *S*1_, are updated according to the equation

$$ {\displaystyle \begin{array}{c}{Q}_{MF\mid S1}\left(a,t+1\right)={Q}_{MF\mid S1}\left(a,t\right)\\ {}+\alpha \left[{Q}_{MF\mid S1}\left(a,t\right)-{Q}_{MF\mid S2}\left({p}_x,t\right)\right]\\ {}+\alpha \lambda \left[r(t)-{Q}_{MF\mid S2}\left({p}_x,t\right)\right],\end{array}} $$

where *λ* is the eligibility trace parameter, *α* is the learning rate and *r*(*t*) the reward received in that trial. To simulate forgetting, *Q* values for the non-chosen actions or unpresented stimuli were decayed by multiplying them by (1 - *α*) (Lau & Glimcher, 2005).

The *model-based mechanism* takes both the instructed and experienced transition structure to construct an internal model of the different task conditions. The cumulative integration and updating of the experienced state-action transitions allows for estimating the underlying model of the environment based on the instructed transition probabilities. Because participants in our study were confronted with both a predictable (75% and 25%) and a random condition (50%) in each block resulting in two distinct internal models, we had to model explicitly the decision process by which participants determined which of the sets was associated with the predictable or random internal model, respectively. This was done by using a higher-level evaluation mechanisms described in previous studies (Daw et al., 2011; Gillan et al., 2015). This process counts the numbers of set-specific transitions from action *a*_*x*_ to second-stage picture *p*_*y*_ for each set. On each trial, it then calculates the differences between counters representing the common and rare assignments within each set. The absolute value of this difference reflects the predictability of a set (because higher values indicate that some transitions are more frequent than others), and the set with the higher absolute difference is identified as the predictable condition. The same counters are then used for determining the direction of the transition structure. For the predictable internal model, the mechanism chooses between two possibilities when presented with first-stage stimulus pair: (1) *P*(*p*_*A*_| *a*_*A*_) = 0.75, P(*p*_*B*_| *a*_*B*_) = 0.75, or (2) *P*(*p*_*A*_| *a*_*A*_) = 0.25, P(*p*_*B*_| *a*_*B*_) = 0.25), with P(*p*_*A*_| *a*_*B*_) = 1 − P(*p*_*A*_| *a*_*A*_) and P(*p*_*B*_| *a*_*A*_) = 1 − P(*p*_*B*_| *a*_*B*_), according to whether the internal transition counter had detected more transitions to *p*_*A*_ following *a*_*A*_ plus *p*_*B*_ following *a*_*B*_ or more transitions to *p*_*B*_ following *a*_*A*_ plus *p*_*A*_ following *a*_*B*_. For the random internal model, the probabilities *P*(*p*_*A*_| *a*_*A*_) = 0.5, P(*p*_*B*_| *a*_*B*_) = 0.5 were applied.

At the second stage, model-based RL coincides with the TD learning algorithm described above, because *Q*_*MF* ∣ *S*2_(*p*_*x*_, *t*) is an estimate of the received reward *r*(t). Estimates for the first-stage model-based values are defined as a mixture of both transition and reward estimates using the Bellman Equation (Bellman, 1957):

$$ {Q}_{MB\mid S1}\left({a}_j,t\right)=P\left({p}_A|{a}_j\right){Q}_{MF\mid S2}\left({p}_A,t\right)+P\left({p}_B|{a}_j\right){Q}_{MF\mid S2}\left({p}_B,t\right) $$

To connect Q values to participants’ choices, a net value which is the weighted combination of the model-free and model-based first-stage action values, *Q*_*net*_(*a*, *t*) = *ωQ*_*MB*_(*a*, *t*) + (1 − *ω*)*Q*_*MF*_(*a*, *t*) was calculated. The model-basedness parameter *ω* approaches 1 if the model-based mechanism is predominant and approaches 0 when the model-free mechanism is predominant. After joining together model-free and model-based action values, the resulting net action values were converted into action probabilities using a softmax function,

$$ P\left({a}_t=a\right)=\frac{\exp \left(\beta \ast {Q}_{net}\left(a,t\right)+\rho \ast rep(a)\right)}{\sum_{a\prime}\exp \left(\beta \ast {Q}_{net}\left(a^{\prime },t\right)+\rho \ast rep\left(a^{\prime}\right)\right)}\kern0.5em , $$

where the inverse temperature parameter *β* guides the stochasticity of the choices and the perseveration parameter *ρ* captures choice perseveration (*ρ* > 0) or switching (*ρ* < 0) (Lau & Glimcher, 2005). The indicator function *rep*(*a*) takes the value 1 if action *a* is the same as that in the last trial of the same set, and zero otherwise.

Using Markov chain Monte Carlo (MCMC) sampling, we estimated the free parameters of multiple Bayesian hybrid RL models for each set, block and participant individually. Additionally, we fit group-level distributions for some models. All parameters were held constant during the blocks. Comparison between the different models via the Watanabe-Akaike Information Criterion (WAIC, Vehtari & Gelman, 2014; Watanabe, 2010) indicated that the best fitting model contained five general parameters (*α*, *λ*, *β*, *ω*, *ρ*) in a hierarchical framework. Values for each set and participant were derived from a single parameter-specific hyperparameter distribution. Reported parameter values represent the mean of their estimated distributions. Models with reduced parameter space and freedom led to higher WAIC scores indicating worse fits. For the subsequent electrophysiological analyses, RPEs were derived by feeding the estimated parameters back into the same model that was used to calculate the model parameters.

### Electrophysiological recordings and ERP analysis

Throughout the experiment, participants were seated comfortable in a dimly lit room. The electroencephalogram (EEG) was recorded using a BIOSEMI Active-Two system (BioSemi, Amsterdam, The Netherlands) with 64 Ag-AgCl electrodes placed according to the extended International 10-20 EEG system, as well as the left and right mastoid. The CMS (common mode sense) and DRL (driven right leg) electrodes were used as reference and ground electrodes. Vertical and horizontal electrooculogram (EOG) were recorded from electrodes above and below the right eye and on the outer canthi of both eyes. All electrodes were offline re-referenced to averaged mastoids. EEG and EOG were continuously recorded at a sampling rate of 512 Hz.

EEG data were band-pass filtered to exclude frequencies below 0.1 Hz and above 40 Hz and divided into epochs from 1,000 ms before to 1,500 ms after feedback onset. Baseline activity from 200 ms before feedback onset was removed. Bad channels were interpolated using spherical spline interpolation if they met the joint probability criterion (threshold 5) as well as the kurtosis criterion (threshold 5) in EEGLAB’s channel rejection routine. Epochs were excluded whenever neural activity in a channel deviated more than ±300 μV from the epoch mean. This criterion was not applied to those channels that are typically contaminated by blinks (Fp1, Fpz, Fp2, AF7, and AF8) as this activity was corrected later. In a next step, an infomax-based independent component analysis (ICA, Bell & Sejnowski, 1995) implemented in EEGLAB was conducted. After visual inspection of the derived independent components, those that represented eye blinks and muscular artifacts were identified and removed from the data. Furthermore, blocks in which participants did misjudge the predictable and random sets were excluded from the analyses (24.5% of all trials^2^). This was done to prevent a biasing effect of incorrectly identified condition mappings. The remaining epochs were averaged separately for each participant and condition. On average, this resulted in the following numbers of artifact-free trials in the respective feedback conditions: 57.9 (*SD* = 21.22) for win/expected/predictable, 30.2 (*SD* = 10.64) for loss/expected/predictable, 14.3 (*SD* = 5.04) for win/unexpected/predictable, 23.2 (*SD* = 9.87) for loss/unexpected/predictable, 40.9 (*SD* = 16.81) for win/expected/random, 43.5 (*SD* = 13.63) for loss/expected/random, 19.6 (*SD* = 8.06) for win/unexpected/random and 19.2 (*SD* = 7.49) for loss/unexpected/random.

Time-frequency measures were computed by multiplying the fast Fourier transformed (FFT) power spectrum of the single-trial EEG data with the FFT power spectrum of a set of complex Morlet wavelets. These wavelets are defined as a family of Gaussian-windowed complex sine waves according to ${e}^{-i2\pi tf}{e}^{-{t}^2/2\sigma 2}$, with the time *t*, the frequency *f* (increasing from 1 to 50 Hz in 50 logarithmically spaced steps) and the width of each frequency band *σ* which was set according to 4/2*πf*. Power was normalized by conversion to a decibel (dB) scale and using a baseline from -300 to -200 prior to the onset of feedback (see Cavanagh, 2015).

In line with previous studies (Chase et al., 2011; Frank, Woroch, & Curran, 2005; Holroyd, Nieuwenhuis, Yeung, & Cohen, 2003; Yeung & Sanfey, 2004), FRN amplitudes were quantified using peak-to-peak measures at electrode FCz. To allow reliable peak amplitude estimation, a 15 Hz low-pass second-order Butterworth filter was applied (Frank et al., 2005). For each participant, the filtered data were split into the conditions of interest and averaged. FRN amplitudes were determined by 1) identifying the most negative peak within a time window of 200-350 ms after feedback onset, and 2) subtracting the average of the preceding and succeeding positive peaks. The preceding and succeeding peaks were quantified as the most positive deflections in time windows 100 ms before and after the FRN peak, respectively. Following Chase et al. (2011), if the maxima were on the edge of the window, the size of the window was stepwise widened by 10 ms up to 300 ms. The averaged empirical latencies ranged between 74 and 287 ms for the preceding peaks, between 184 and 340 ms for the FRN peaks, and between 244 and 490 ms for the succeeding peaks. Theta band power (4-8 Hz; Cavanagh, 2010) was measured as the mean amplitude in a time window of 200-400 ms after feedback presentation (Cavanagh et al., 2010; Sambrook & Goslin, 2014) at electrode FCz. The P3 amplitude and the delta band power (1-4 Hz; Cavanagh, 2015) were measured as the mean amplitude/power in a time window of 300-500 ms after feedback presentation at electrode Pz (Cavanagh, 2015; Chase et al., 2011; San Martín, 2012). Time domain and frequency domain components were averaged separately for each participant and condition. For both ERP and time-frequency analyses, we chose an analysis design that compares losses and wins of equal probability and thus expectancy (Holroyd et al. 2009). Wins after transitions from high-probability reward stimuli (70%) and losses after transitions from low-probability reward stimuli (70%) at the second stage were assigned to the expected outcome condition. Wins after low-probability reward stimuli (30%) and losses after high-probability reward stimuli (30%) at the second stage were assigned to the unexpected outcome condition. Expectancy was thus solely based on the fixed reward structure, which was analogous for the predictable and random conditions. We applied repeated measures ANOVAs involving the variables condition (predictable, random), expectancy (expected, unexpected) and valence (win, loss) for each dependent measure (FRN amplitudes, theta power, P3 amplitudes, delta power).

To analyze the relationship between EEG activity and RPEs in our computational model, we quantified single-trial amplitudes of our neural measures using the identical measures as in the analyses for the averaged EEG activity. Single-trial FRN amplitudes were quantified using the peak-to-peak method. Single-trial P3 amplitudes were quantified using the mean amplitude in the time window of 300-500 ms after feedback presentation. Single-trial theta power and single-trial delta power was quantified by averaging across the respective time windows (theta: 200-400 ms; delta: 300-500 ms) and frequency spectra (theta: 4-8 Hz; delta: 1-4 Hz) at the respective electrodes. Given the ongoing debate about whether the FRN and theta power reflects signed or unsigned RPEs (Chase et al., 2011), separate regression analyses for positive and negative RPEs were calculated, and absolute values of model RPEs were used. Accordingly, we expected similar signs for regression slopes of positive and negative feedback if this activity reflects an unsigned RPE. Slopes were tested against zero using one-sample *t*-tests and were entered into an ANOVA involving the variables valence (positive RPEs, negative RPEs) and condition (predictable, random).

## Results

### Behavioral data

In a first analysis, we investigated to which extent participants were able to improve their choice behavior via learning in the predictable and random conditions. For the predictable condition, an obvious measure of correct task performance is the proportion of first-stage responses leading to the high reward second-stage stimulus via a common transition. Figure 1D shows that this proportion reaches about 80% at the end of the block. However, such a measure is not applicable to the random condition in which no “correct” first-stage response can be defined. To allow for a comparison of the two conditions, we therefore considered the bonus obtained in each condition. We found the mean bonus per block in the predictable condition (*M* = 0.16 €, *SD* = 0.16) to be significantly higher than in the random condition (*M* = 0.02 €, *SD* = 0.17), *t*(28) = 3.88, *p* < 0.001. Moreover, only in the predictable condition participants obtained a bonus that exceeded chance level (which is zero), *t*(28) = 5.39, *p* < 0.001, whereas this was not the case for the random condition, *t*(28) = 0.51, *p* = 0.61. As expected, these results show that participants could successfully improve choice behavior by learning in the predictable condition but not in the random condition.

To investigate whether participants show hallmarks of both model-free and model-based learning in the predictable condition, we analyzed participants’ stay and switch behavior at the first decision stage as a function of feedback presented on the previous trial from the same condition. As a solely reward-driven learning mechanism, model-free learning is characterized by switch behavior following losses and stay behavior following wins (main effect of reward type). In contrast, model-based learning additionally relies on the task’s transition structure guiding behavior. Therefore, model-based learning in the predictable condition is indicated by a distinct choice pattern that advocates switches after losses following common transitions and after wins following rare transitions but stays after wins following common transitions and after losses following rare transitions (interaction between reward and transition type). The left column of Table 1 shows the results of the logistic regression analysis for the predictable condition. Consistent with previous studies using related learning tasks (Daw, 2011; Gillan et al., 2015; Otto, Gershman, Markman, & Daw, 2013), we found that participants show both hallmarks of model-free learning (main effect of reward type) and model-based learning (interaction between reward and transition type). Figure 1C (left part) shows the typical pattern indicating a mixture of model-free and model-based control. While stay probabilities were generally higher following wins (model-free control), this effect was modulated by an interactive pattern indicating a reduced link between wins and stay behavior following rare transitions (model-based control). Because model-free and model-based behavior cannot be distinguished in the random condition, we analyzed these data in a separate analysis. A simplified model without the variable transition type showed an effect of reward type, indicating an increased stay probability following wins (Figure 1C, right part). Together, these analyses demonstrate that behavior in the predictable condition involved both model-free and model-based aspects, whereas behavior in the random condition indicated that participants utilize feedback for behavioral adaptation even though this cannot lead to an increased reward in this condition.

**Table 1:** Results of the logistic regression predicting stay probabilities for the predictable and random condition

Coefficient	Predictable		Random	
	F Value	*p* value	F Value	*p* value
(Intercept)	**129.14**	**<0.001**	**67.00**	**<0.001**
Reward	**43.47**	**<0.001**	**30.98**	**<0.001**
Transition	0.004	0.95	-	-
Reward x Transition	**22.31**	**<0.001**	-	-

### Averaged EEG data

We were now interested in the neural correlates of feedback processing. Crucially, we expected to find differential effects of expectancy and valence across conditions for those neural components that are influenced by the type of internal model. More specifically, we hypothesized that expectancy and valence effects for these components are solely or more strongly observable in the predictable condition as only this condition allows for a feedback-based adaptation of first-stage decisions.

**Fig. 2:**
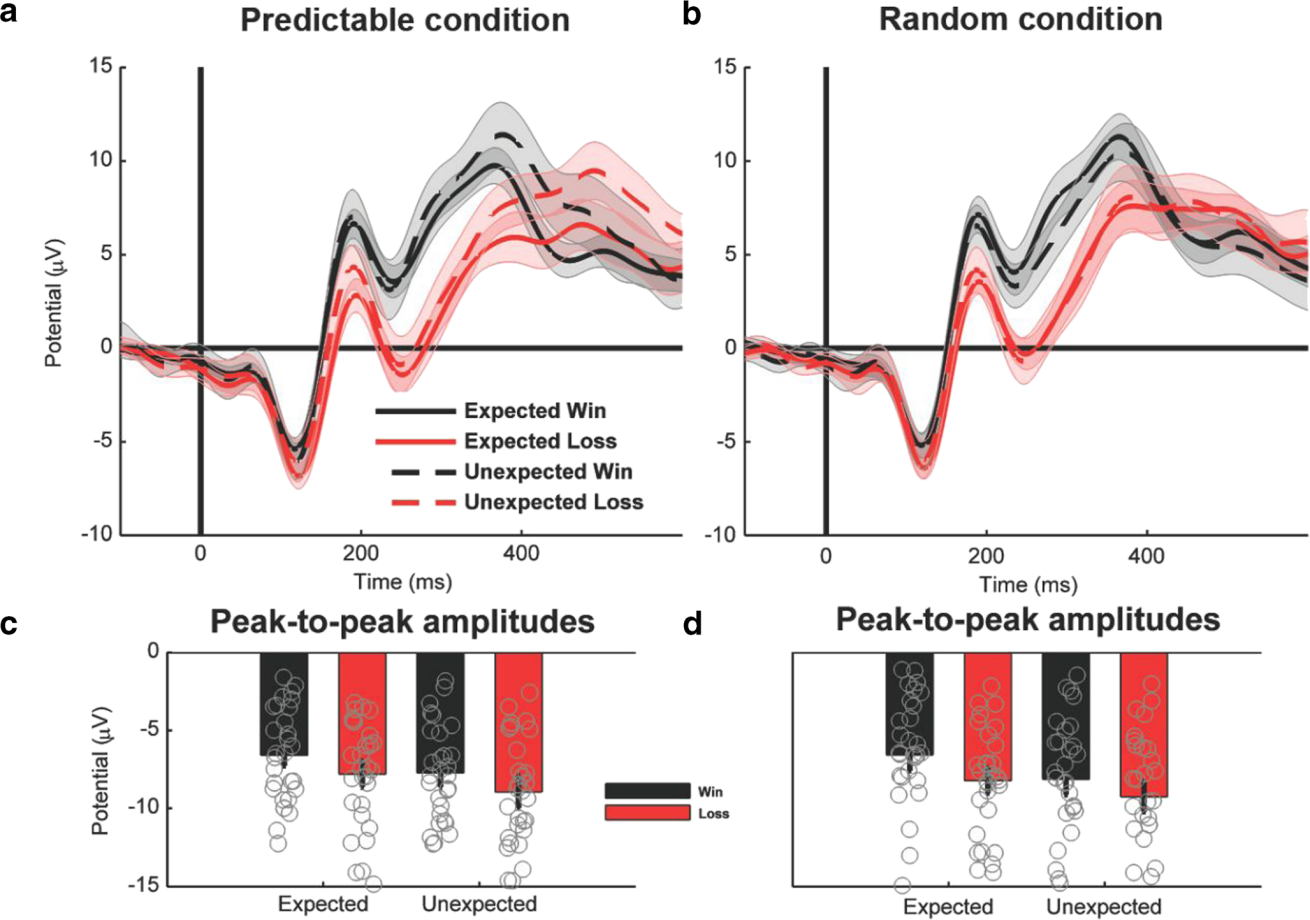
Feedback-locked time-domain activity at electrode FCz**. a, b:** Grand average waveform for the predictable and the random conditions*.* Shaded areas show the 95% confidence intervals. **c, d:** Peak-to-peak amplitudes. Gray circles indicate the amplitudes for the individual subjects

**Fig. 3:**
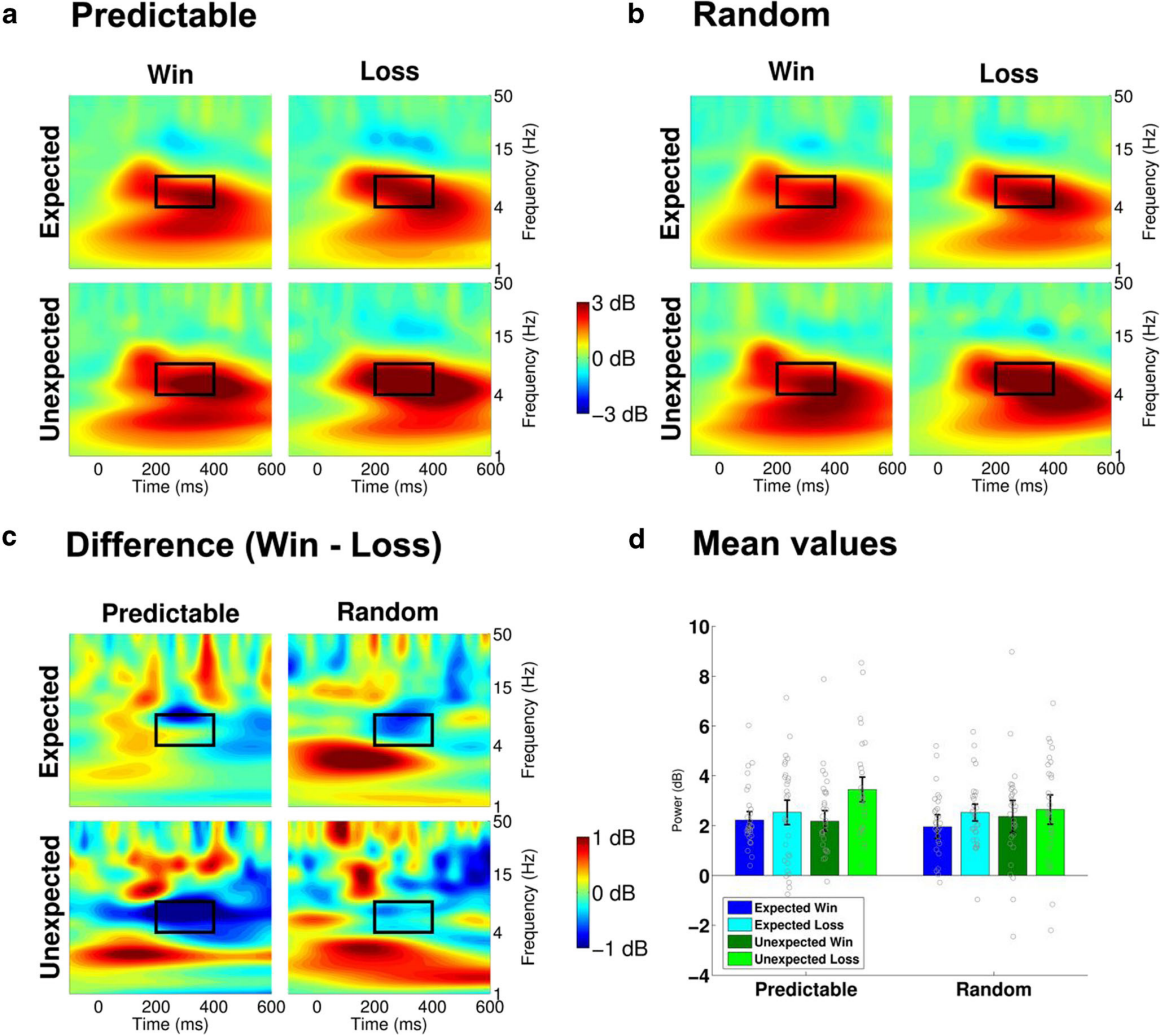
Feedback-locked theta frequency neural activity at electrode FCz**. a, b: **Estimated power for the predictable and the random conditions. The black rectangle specifies the time window (200-400 ms) and frequency window (4-8 Hz, theta) of interest. **c:** Logarithmic frequency scaling of the difference between losses and wins for each condition and expectedness. **d:** Mean power values in the 200-400 ms time window and 4-8 Hz frequency window. Gray circles indicate the power values for the individual subjects

Analyzing *FRN amplitudes* at electrode site FCz (Figure 2) using peak-to-peak measures, we found a significant main effect of expectancy, *F*(1, 28) = 15.77, *p* < 0.001, and a marginally significant main effect of valence, *F*(1, 28) = 3.79, *p* = 0.062, showing more negative amplitudes for unexpected feedback and for losses compared to expected feedback and wins. There were no further significant main effects or interactions (*F*s < 0.28, *p*s > 0.600). Analyzing *theta band activity* at the same electrode site (Figure 3), we found a marginally significant main effect of valence, *F*(1, 28) = 3.42, *p* = 0.075, with more theta power for losses than for wins. Again, this effect did not interact significantly with any other variable (*F*s < 2.13, *p*s > 0.16). In addition, we obtained a significant main effect of expectancy, *F*(1, 28) = 7.72, *p* = 0.010, indicating higher power for unexpected outcomes compared with expected outcomes.

Taken together, we obtained similar results for the FRN and theta band power regarding valence and expectancy modulations, although some of these effects reached only marginal significance. Crucially, none of these effects differed between predictable and random conditions, which suggests that the FRN and theta are unaffected by the internal model.

**Fig. 4:**
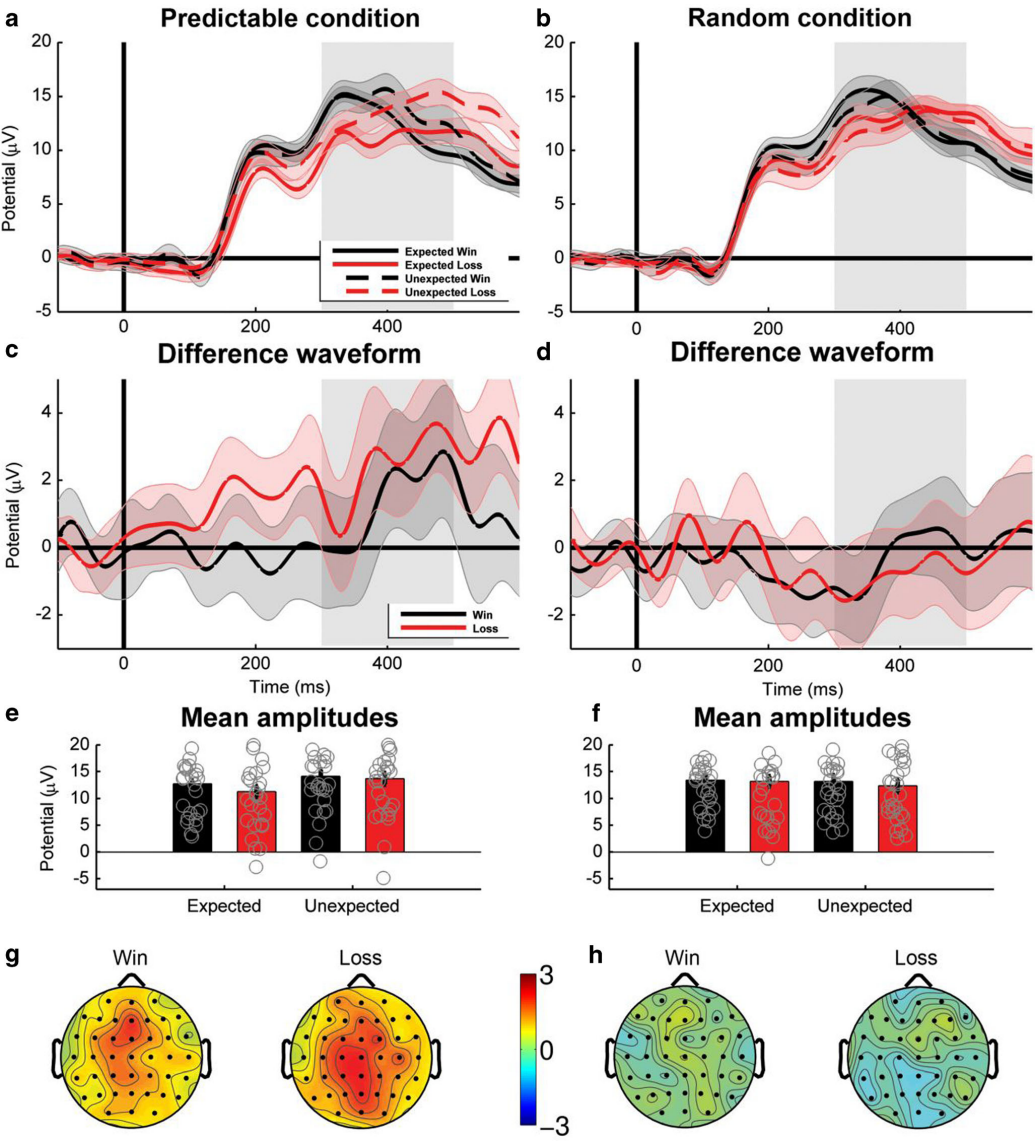
Feedback-locked time-domain neural activity at electrode Pz**. a, b:** Grand average waveform for the predictable and the random conditions. Shaded areas show the 95% confidence intervals. **c, d:** Difference waves of the expectancy effect for the predictable and the random conditions, calculated as unexpected minus expected. Shaded areas show the 95% confidence intervals. **e, f:** Mean amplitudes in the 300-500 ms time window. Gray circles indicate the mean amplitudes for the individual subjects. **g, h:** Topographies of the difference wave between unexpected and expected for each condition and valence 300-500 ms after feedback onset

**Fig. 5:**
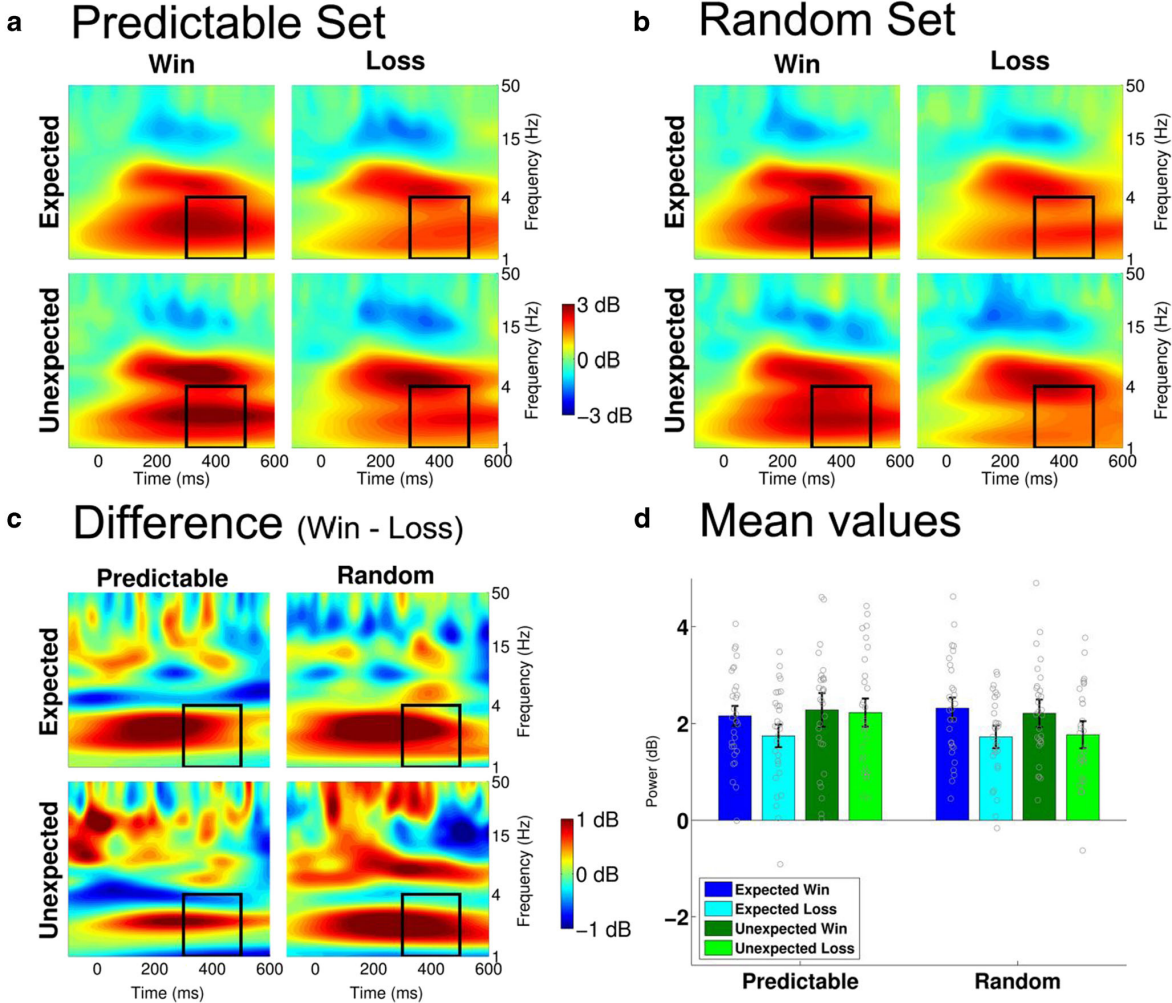
Feedback-locked delta frequency neural activity at electrode Pz**. a, b:** Estimated power for the predictable and the random conditions. The black rectangle specifies the time window (300-500 ms) and frequency window (1-4 Hz, delta) of interest. **c:** Logarithmic frequency scaling of the difference between losses and wins for each condition and expectedness. **d:** Mean power values in the 300-500 ms time window and 1-4 Hz frequency window. Gray circles indicate the power values for the individual subjects

Analyzing *P3 amplitudes* at electrode site Pz (Figure 4), we found a marginally significant main effect of expectancy, *F*(1, 28) = 4.15, *p* = 0.051, which was qualified by a significant interaction between condition and expectancy, *F*(1, 28) = 13.30, *p* = 0.001. Separate analyses for the two conditions revealed that unexpected outcomes led to a more positive waveform than expected outcomes for the predictable condition, *F*(1, 28) = 14.43, *p* < 0.001, but not for the random condition, *F*(1, 28) = 1.35, *p* = 0.26^3^. Analyzing *delta band activity* at the same electrode (Figure 5), we found a significant main effect of reward, *F*(1, 28) = 7.90, *p* = 0.009, yielding higher power for wins compared to losses. Furthermore, we found a marginally significant interaction between condition and expectancy, *F*(1, 28) = 4.11, *p* = 0.052. Again, unexpected outcomes were associated with more activity than expected outcomes for the predictable condition, *F*(1, 28) = 5.82, *p* = 0.023, but not for the random condition, *F*(1, 28) = 0.05, *p* = 0.81.

Taken together, both the P3 and delta band activity showed a congruent pattern of stronger modulation of expectations for the predictable condition compared to the random condition. This suggests that these differences are linked to the different internal models in the two conditions. Because only the predictable condition allows for feedback-based adaptation of first-stage decisions, signatures of (model-based) feedback evaluation, such as expectancy effects, are increased in the predictable condition.

### Model-based analysis of single-trial EEG data

After finding first neural evidence for the differential effects of internal models in the predictable and random conditions, we fit a computational reinforcement learning model, which explicitly models the effects of these internal models on behavior. The predictable internal model was implemented using the predictable transition structure in order to estimate the first stage actions, leading to standard model-based behavior (Daw et al., 2011; Gillan et al., 2015) as demonstrated in the behavioral analyses. The random internal model was implemented using the random transition structure, which introduced stochastic choice behavior as an alternative to model-free action selection. Table 2 shows the estimated parameter values in the predictable and random conditions as well as the results of *t*-tests of the condition difference.

**Table 2:** Results of the comparison between the parameters derived from the computational model

Parameter	Bounds	Predictable^1^	Random^1^	*t*-value^2^
Learning rate	[0,1]	0.21(0.04)	0.21(0.04)	0.74
Eligibility trace	[0,1]	0.96(0.01)	0.96(0.01)	0.27
Inverse temperature	[0, ∞]	1.95(0.67)	1.63 (0.51)	2.59*
Model-basedness	[0,1]	0.33(0.07)	0.29(0.08)	-^3^
Perseveration	[-∞,∞]	0.73(0.29)	0.76(0.28)	-0.63

Note. We fit a hierarchical Bayesian model using MCMC sampling with set-specific free parameters. We ran 4 chains with 2,000 iterations (1000 warm-up). Rhat < 1.1.

^1^Mean (standard error)

^2^df = 28, *p < 0.05, **p < 0.01

^3^No statistical test of the model-basedness parameter was applied, because this parameter possibly has a different meaning in the two conditions. While it refers to the contribution of model-based control in the predictable condition, it represents an additional source of noise (i.e., randomness) in the random condition

In a next step, we used the estimated RPEs from the computational model to inform our analysis of the neural data on a single-trial level. Formally, the model does not distinguish between model-based and model-free control at the time of feedback processing as model-based learning coincides with model-free learning at the feedback stage. However, it is conceivable that the feedback-related RPE in the model is reflected differently in neural activity based on the internal model. Our hypotheses were quite similar to those in the averaged EEG results section. We expected that components sensitive to the internal model correlate with RPEs more strongly in the predictable than in the random condition. In all analyses, we used the absolute values of RPEs independent of sign, and thus feedback valence. If the sign of the slopes remains the same in both valence conditions, then the underling activity reflects an unsigned RPE. If, however, the sign of the slopes differs as a function of valence, the underlying activity reflects a signed RPE.

**Fig. 6:**
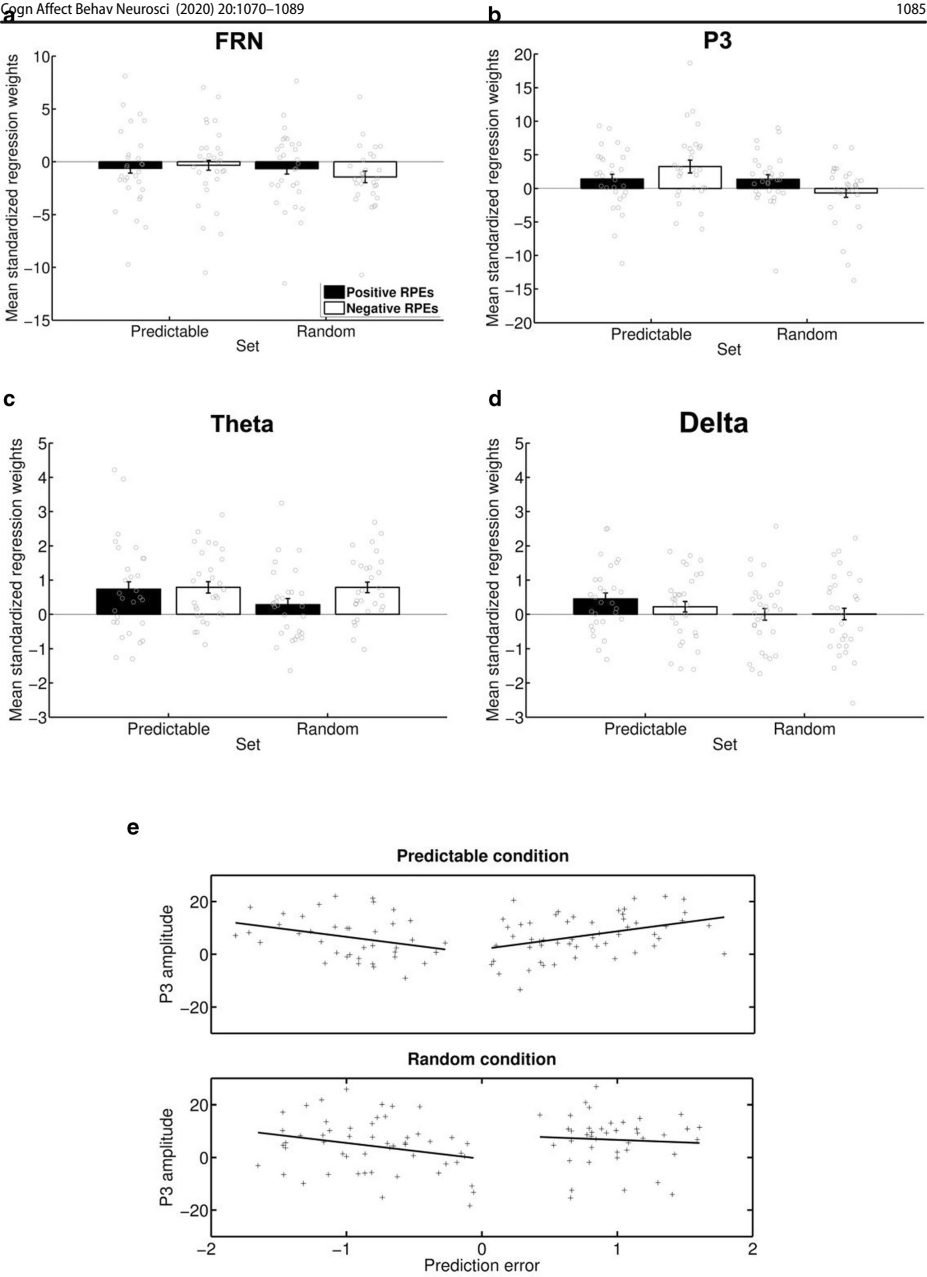
Mean standardized regression weights for the relationship between absolute reward prediction error estimates and single-trial neural activity**.** Gray circles indicate regression weights for the individual subjects. **a:** FRN activity was estimated via peak-to-peak measures at electrode FCz. **b:** P3 activity was estimated via averaging at electrode Pz in the 300-500 ms time window. **c:** Theta activity was estimated via averaging at electrode FCz in the 200-400 ms time window and 4-8 Hz frequency window. **d:** Delta activity was estimated via averaging at electrode Pz in the 300-500 ms time window and 1-4 Hz frequency window. **e:** Representative data from participant 16 for single-trial regression between reward prediction error estimates and P3 amplitudes. Note that for the results reported we used absolute reward prediction errors

Regressions involving RPEs and *single-trial FRN amplitudes* (Fig. 6A) revealed a significant negative overall slope, *t*(28) = 2.57, *p* = 0.016, indicating that trials with larger RPEs show larger (negative) FRN amplitudes. An ANOVA with the variables condition and valence revealed no significant effects (*F*s < 2.03, *p*s > 0.165). Regressions involving RPEs and *single-trial theta band activity* (Figure 6C) showed a positive overall slope, *t*(28) = 6.08, *p* < 0.001, suggesting that trials with larger RPEs show larger theta band power. But again, no significant effects were obtained in the ANOVA (*F*s < 1.92, *p*s > 0.17).

Regressions involving RPEs and *single-trial P3 amplitudes* (Figures 6B and 6E) revealed a significant main effect of condition in the ANOVA, *F*(1, 28) = 4.25, *p* = 0.049, indicating that in the predictable condition, the coupling between single-trial amplitudes and RPEs was stronger compared with the random condition. Qualifying this finding, a significant positive slope was observed only for the predictable condition, *t*(28) = 2.32, *p* = 0.023, but not for the random condition, *t*(28) = 0.63, *p* = 0.53, revealing that P3 amplitudes scaled with absolute RPE magnitude only in the predictable condition. However, a significant interaction between condition and valence, *F*(1, 28) = 6.02, *p* = 0.020, indicates that this positive relationship in the predictable condition is particularly high (and significant only) for negative feedback, *t*(28) = 3.30, *p* = 0.003. Regressions involving RPEs and *delta band power* (Figure 6D) revealed no significant overall slope, *t*(28) = 1.46, *p* = 0.16, and only a marginally significant effect of condition, *F*(1, 28) = 3.28, *p* = 0.081. While positive slopes were numerically higher in the predictable condition, they still failed to reach significance, *t*(28) = 1.43, *p* = 0.16.

Taken together, we found significant correlations between RPEs in our model and three of our four types of feedback-related brain activity. As predicted, FRN amplitudes and theta band power correlated with RPEs independent of condition, suggesting that these components are independent of the internal model. In contrast, P3 amplitudes correlated with RPEs only for the predictable condition (and only for negative feedback), suggesting that this reflects in-depth feedback processing only under a predictable but not a random internal model.

## Discussion

Following current psychological theories of reinforcement learning, decision making under uncertainty results from the interaction of two distinct reinforcement learning mechanisms, namely habitual model-free learning and goal-directed model-based learning (Balleine & O’Doherty, 2010; Daw et al., 2005). While model-free learning relies on previous experience with a task, model-based learning uses explicit knowledge about environmental contingencies to construct internal models which allow planning and flexible behavior. The goal of the present study was to investigate how different internal representations of the environment can influence behavioral adaptation and neural feedback processing. In order to answer this question, we implemented a modified version of the two-stage Markov decision task that employed two sets with different transition structures (see also Eppinger et al. 2017). In the predictable condition, transition probabilities were highly differentiated which should favor the application of an internal model reflecting this predictable structure. In the random condition, transition probabilities were fixed at chance which should favor the application of an internal model reflecting this random structure. Crucially, however, the reward structure, that is, the conditional probability of a win given a specific stage-two stimulus, was the same for predictable and random conditions. By contrasting the predictable condition with the random condition, we were able to specifically pinpoint modulations of time and frequency-domain feedback-locked components that reflect feedback processing under the different internal models. As an important prerequisite for further analyses, we replicated the prominent behavioral finding of a mixture of model-free and model-based RL in the predictable condition (Daw et al., 2011; Gillan et al., 2015), which suggests the application of a predictable internal model. Additionally, in the random condition, we also found a signature of win-stay/lose-shift behavior, thus illustrating that feedback was used to adapt behavior even though this could not improve performance.

In the time domain, ERP findings clearly indicate that the FRN and P3 component reflect separable neural mechanisms of feedback processing with different sensitivity to the underlying internal model in use. In line with our predictions, the feedback-locked ERP analysis showed that the neural pattern reflected in the P3 was clearly differentiated across conditions. Consistent with previous findings from trial-and-error tasks (Donaldson, Ait, Sebastien, & Foti, 2016; Hajcak et al., 2005; Holroyd, Krigolson, Baker, Lee, & Gibson, 2009), we found higher P3 amplitudes for unexpected outcomes compared to expected outcomes. Crucially, this effect was only evident in the predictable condition, in which a predictable internal model was applied, but was absent in the random condition, in which a random internal model was applied. We conclude that the feedback-locked P3 is sensitive to the environmental contingencies as represented by the internal model.

Concerning the FRN effect, our analysis showed strong similarities between conditions. In line with previous findings, the FRN was strongly modulated by the expectancy of the outcomes with more pronounced effects after unexpected feedback compared to expected feedback (Eppinger, Kray, Mock, & Mecklinger, 2008; Holroyd & Coles, 2002; Walsh & Anderson, 2011a, 2011b; but see San Martín, 2012). Interestingly, the FRN effect (i.e., a larger negativity following losses as following wins) was evident in our data but failed to reach significance. This is surprising, because the FRN effect seems to be the most widely reported and stable effect associated with this component (San Martín, 2012; Walsh & Anderson, 2012). It could reflect that the absolute amplitude of the FRN peak represents an unsigned prediction error. Unsigned prediction errors should be similar for positive and negative feedback in our paradigm due to the fact that (conditional) expectancy is perfectly balanced across the two types of outcome. Summing up, the ERP results of the present study suggest that while the FRN component was unaffected by the type of internal model, the feedback-locked P3 was strongly influenced by the internal models.

The results from the time domain were complemented and almost paralleled by comparable effects in the frequency domain. By showing a very similar pattern as the P3 amplitudes with stronger expectancy effects in the predictable condition than in the random condition, centroparietal delta band power was clearly affected by the environmental contingencies represented in the internal model. In contrast, theta band power exhibited no such effect of task condition. Comparable to the FRN, a strong expectancy effect and only a weak valence effect in frontocentral theta did not differ between the predictable and the random condition, suggesting an insensitivity of the theta band to the internal model. Together, feedback-related brain activity in the frequency domain shows a very similar dichotomy as in the time domain with only delta-band power being sensitive to the type of the internal model.

To further substantiate our findings, we applied a computational modeling analysis, which showed that a considerable amount of model-based control was exerted in the predictable condition whereas the random condition was characterized by stochastic choice behavior. Most important, this computational model allowed for investigating the relationship between estimated RPEs and feedback-related brain activity on a single-trial level. Although the model did not distinguish between model-free and model-based feedback processing, we hypothesized that while the RPEs should be reflected in FRN amplitudes and theta band activity irrespective of experimental condition and internal model, P3 and delta band activity should be sensitive to the environmental contingencies dictated by the internal model at hand. Our results provided further support for this assumption: FRN amplitudes and theta band activity correlated with RPEs in both predictable and random conditions, suggesting that RPEs are reflected in these fronto-central components (Holroyd and Coles 2002; Cavanagh et al. 2010) but are independent of the internal model. Interestingly, both the FRN and theta band activity in our data appear to reflect an unsigned RPE (Alexander & Brown, 2011; Sambrook & Goslin, 2015), which is line with our interpretation of the FRN results as discussed above. In contrast, P3 amplitudes correlated with RPEs in predictable conditions but not in random conditions, suggesting that RPEs are reflected in this component dependent on the specific internal model at hand.

Taken together, our results provide support for the idea that the neural components of feedback processing are differentially modulated by different internal models. On the one hand, some components, such as the FRN in our study, are unaffected by representations of environment contingencies but still capture basic outcome dimensions (e.g., valence and expectancy of feedback or aspects of model-free learning, i.e., RPEs). On the other hand, some components, such as the P3 in our study, are strongly affected by internal models. In line with previous research (Nieuwenhuis, Holroyd, Mol, & Coles, 2004; Wu & Zhou, 2009), this suggests that the FRN is still involved in learning of the reward structure (e.g., by representing an RPE), but that higher-order evaluations such as secondary stimulus features and learning context are not taken into account. In contrast, the P3 reflects such higher-order integration of secondary stimulus features and learning context to flexibly learn the reward structure and even set up for upcoming behavioral adaptation (Bernat et al., 2015). Our results also are compatible with the view that the P3 rather than the FRN is related to the initiation of behavioral adaptation. While some studies reported that the FRN is also associated with behavioral adaptation (Cohen & Ranganath, 2007), recent findings suggest that the processes underlying the generation of the FRN and the related reward positivity are dissociable from the processes responsible for behavioral adaptation which are linked to the P3 (Chase et al., 2011; Cockburn & Holroyd, 2018; Yeung, Holroyd, & Cohen, 2005; for a discussion see San Martín, 2012).

An important question is whether model-based control is involved only in the predictable condition or also in the random condition. A core idea of our study is that participants form internal models (i.e., explicit representations of transition probabilities) in both conditions, and that the different internal models are responsible for the differential patterns in feedback-locked brain activity. However, the exact role of model-based control in these effects are unclear. From our view, there are two possibilities, which mainly differ in their definition of model-based control. First, if model-based control is narrowly defined as translating knowledge about predictable transitions into expectations that inform RL, then only the predictable condition involves model-based control. In this case, contrasting the predictable and random conditions in our paradigm corresponds to a comparison between a condition with model-based control and a condition without model-based control. Second, if model-based control is wider defined as any goal-directed influence of explicit knowledge on RL, then the computational mechanism in our model that introduces stochastic choice behavior (i.e., randomness) in the random condition also could be interpreted as model-based control. In this case, our conditions do not differ regarding whether model-based control is exerted but regarding the type of model-based control.

The latter possibility receives support from the observation that stochastic choice behavior can be viewed as an adaptive and goal-directed strategy in certain tasks. In a recent study (Tervo et al., 2014), rats only received reward if their decision differed from the decision of a competitor on the same trial. For choice patterns of specific competitors, rats were able to maximize reward by switching to stochastic choice behavior. Although it is unclear whether this strategy was driven by an internal model (Tervo et al. denied this idea), these findings provide support for the idea that randomness can be goal-directed and adaptive. Interestingly, the switch towards stochastic behavior in this study was mediated by noradrenergic input from the locus coeruleus (LC) to the anterior cingulate (Tervo et al., 2014)—two structures with a strong link to the neural activity investigated in our study. The FRN is hypothesized to originate from the anterior cingulate (Holroyd & Coles, 2002; Miltner, Braun, & Coles, 1997) and the P3 is linked to noradrenergic activity via the LC-P3 hypothesis (Nieuwenhuis, 2011; Nieuwenhuis et al., 2005). This raises the possibility that both components could play a role in arbitrating between decision strategies.

But how is the model-based mechanism able to keep track of the environmental contingencies and generate distinct models which enable goal-directed planning? A plausible solution is the additional recruitment of working memory functions. While the updating of working memory representations has frequently been assumed to be reflected in the P3 and delta band activity (Donchin & Coles, 1988; Nieuwenhuis et al., 2005), new evidence suggests that the P3 constitutes a target identification mechanism which is only guided by working memory (Rac-Lubashevsky & Kessler, 2019). Nevertheless, there are two principal ways how working memory could become involved under model-based control. First, information from outcome feedback could be utilized to update the internal world model, thus advancing explicit learning in the model-based mechanism via experience. Evidence for this comes from a variety of neuroimaging and modeling studies (Braver and Cohen 2000; O’Reilly and Frank 2006; D’Ardenne et al. 2012). For example, a recent high-resolution fMRI study showed a correlation between BOLD signals in VTA, a region implicated in generating phasic dopamine signals, and rDLPFC, a region implicated in context encoding and working memory, which suggests that phasic dopamine signals (i.e., outcome information) regulate encoding and updating of context representations in PFC (D’Ardenne et al., 2012). Second, outcome representations in working memory could be used to gate learning in the dopaminergic reinforcement learning system. Evidence for such a top-down gating mechanism comes from studies linking reinforcement learning to working memory capacity and load (Collins, Albrecht, Waltz, Gold, & Frank, 2017; Collins, Brown, Gold, Waltz, & Frank, 2014; Collins & Frank, 2018). For example, Collins et al. (2017) showed that the generation of learning signals was affected by working memory load with stronger RPEs being generated under high working memory load. Eppinger, Walter, Heekeren, and Li, (2013) provided evidence for a link between working memory capacity and the strength of model-based control, and argued that working memory is necessary for the integration of model free learning signals with model-based control. Taken together, this clearly shows the importance of the mechanisms of working memory recruitment and further emphasizes their significance for understanding model-based learning.

Notably, our ERP findings nicely complement a study by Eppinger et al. (2017) investigating the interplay between model-free and model-based decision processes using a rather similar experimental design regarding the transition structure. Although not including the feedback-locked P3 in their analyses, the authors show that the stimulus-locked P3 at the second stage of the Markov task (which required a choice in their study) reflected the integration of both model-free and model-based calculations, whereas the FRN following feedback did not. This finding parallels our results and supports a putative mechanism linking the FRN and the feedback-related P3 to model-free and model-based reinforcement learning. While the FRN seems to reflect a model-free process, more specifically the calculation of a RPE conveyed by the dopaminergic reward system (Holroyd & Coles, 2002), the P3 seems to signal the utilization of such model-free calculations which is dependent on the internal model. While the integration of model-free estimations helps maximizing reward in a predictable environment, this is not the case in a random environment.

Another effort in this direction was reported by a recent EEG study (Sambrook et al., 2018), which reports a method to separate model-free and model-based RPEs in the classic variant of the Markov decision task. The authors showed that neural activity varies with model-free and model-based RPEs at the feedback stage. Moreover, a correlation between both RPEs and early frontal components emphasized the temporal and spatial interplay between learning mechanisms. While the early frontal correlate of model-based RPEs was independent of participants’ model-basedness, late centroparietal activity was strongly modulated by the extent of expressed model-based control (Sambrook et al., 2018). Due to its temporal and spatial characteristics, this late model-based effect is discussed by the authors to originate from neural sources primarily associated with the P3. This interpretation is fully compatible with the present result that the P3 is influenced by the internal model. Both approaches share the notion of a two-fold instantiation of reinforcement learning on the neural level: Early calculation of RPEs and subsequent later utilization, whereas the latter is particularly dependent on model-based control. In conclusion, this calls for the reevaluation and modification of standard reinforcement learning models in order to further elucidate the interplay between the cognitive mechanisms of model-free and model-based learning mechanisms in the human brain.

Taken together, our findings further substantiate the assumption, that the feedback-locked components under investigation reflect different mechanisms of feedback processing (Bernat et al., 2015; Cavanagh, 2015; Hajcak, Moser, Holroyd, & Simons, 2006; Nieuwenhuis et al., 2004). While early frontal components (FRN and theta) are suggested to reflect a first evaluation of outcomes by a model-free RL mechanism, subsequent posterior components (P3 and delta) are supposed to be involved in higher-order evaluations executed or supervised by a RL mechanism that is guided by an internal model. Crucially, our study further suggests that different internal models of the task environment exert control over reinforcement learning only at this latter stage of feedback processing, leading to a selective integration and updating of new information.

## Footnotes

1 We define an internal model as any explicit mental representation of contingencies, here, between first-stage decisions and second-stage stimuli of the Markov decision task. While we assume that the predictable and random conditions lead to the formation of different internal models, this does not necessarily imply that the random condition involves model-based control. We will further discuss this question in the discussion section.

2 These trials were removed to prevent additional noise in the analysis. However, including these trials did not qualitatively change the pattern of results.

3 Figure 4G shows that these expectancy effects have no clear posterior distribution but also reach anterior electrodes. It is therefore possible that our P3 results are generated by two separate positivities: one with an anterior maximum and one with a posterior maximum. Therefore, we also analyzed P3 amplitudes in the 300-500 ms time window at electrode site FCz. We obtained a similar pattern as that at electrode Pz, with a significant main effect of expectancy, *F*(1,28) = 7.50, *p* = 0.011, which was qualified by a significant interaction between expectancy and condition, *F*(1,28) = 5.16, *p* = 0.031. Additionally and in contrast to the posterior P3, we found a significant main effect of valence, *F*(1,28) = 5.49, *p* = 0.026, with more positive amplitudes following wins compared with losses. It seems possible that this pattern from our explorative analysis could be generated by a second, more anterior positivity. However, there is no conclusive evidence in this regard.Visual inspection of Figures 4A and 4B further suggests strong latency variance for the P3 peaks. Analyzing the latencies, measured at the most positive peak in the 300-500 ms time window at electrode Pz, we only found a main effect of valence, *F*(1.28) = 44.98, *p* < 0.001 and expectancy, *F*(1,28) = 8.22, *p* = 0.008, but no significant interactions, especially not with condition. An identical pattern was found at electrode location FCz.
